# Microbiopsy Sampling for Examining Age-Related Differences in Skeletal Muscle Fiber Morphology and Composition

**DOI:** 10.3389/fphys.2021.756626

**Published:** 2022-01-10

**Authors:** Garrett M. Hester, Trisha A. VanDusseldorp, Phuong L. Ha, Kaveh Kiani, Alex A. Olmos, Melody Jabbari, Shania Kalladanthyil, SooBin An, Alyssa R. Bailly, Benjamin E. Dalton, Anton L. Bryantsev

**Affiliations:** ^1^Department of Exercise Science and Sport Management, Kennesaw State University, Kennesaw, GA, United States; ^2^Department of Molecular and Cellular Biology, Kennesaw State University, Kennesaw, GA, United States

**Keywords:** atrophy, muscle quality, microbiopsy, aging, myofiber, C-terminal agrin fragment (CAF), vastus lateralis, immunofluorescence

## Abstract

**Introduction:** The increasingly popular microbiopsy is an appealing alternative to the more invasive Bergström biopsy given the challenges associated with harvesting skeletal muscle in older populations. Parameters of muscle fiber morphology and composition derived from the microbiopsy have not been compared between young and older adults.

**Purpose:** The purpose of this study was to examine muscle fiber morphology and composition in young (YM) and older (OM) males using the microbiopsy sampling technique. A secondary aim was to determine if specific strength is associated with serum levels of C-terminal agrin fragment [CAF; an indicator of neuromuscular junction (NMJ) degradation].

**Methods:** Thirty healthy, YM (*n* = 15, age = 20.7 ± 2.2 years) and OM (*n* = 15, age = 71.6 ± 3.9 years) underwent ultrasound imaging to determine whole-muscle cross-sectional area (CSA) of the vastus lateralis and rectus femoris as well as isometric and isokinetic (60°⋅s^–1^ and 180°⋅s^–1^) peak torque testing of the knee extensors. Microbiopsy samples of the vastus lateralis were collected from 13 YM and 11 OM, and immunofluorescence was used to calculate CSA and proportion of type I and type II fibers.

**Results:** Peak torque was lower in OM at all velocities (*p* ≤ 0.001; *d* = 1.39–1.86) but only lower at 180°⋅s^–1^ (*p* = 0.003; *d* = 1.23) when normalized to whole-muscle CSA. Whole-muscle CSA was smaller in OM (*p* = 0.001; *d* = 1.34), but atrophy was not present at the single fiber level (*p* > 0.05). Per individual, ∼900 fibers were analyzed, and type I fiber CSA was larger (*p* = 0.05; *d* = 0.94) in OM which resulted in a smaller type II/I fiber CSA ratio (*p* = 0.015; *d* = 0.95). CAF levels were not sensitive to age (*p* = 0.159; *d* = 0.53) nor associated with specific strength or whole-muscle CSA in OM.

**Conclusion:** The microbiopsy appears to be a viable alternative to the Bergström biopsy for histological analyses of skeletal muscle in older adults. NMJ integrity was not influential for age-related differences in specific strength in our healthy, non-sarcopenic older sample.

## Introduction

The proportion of older Americans is expected to continue increasing, but the number of very old adults is projected to dramatically increase. For example, the number of adults 65 years and over is expected to nearly double from 49 million in 2018 to 95 million in 2060, whereas the number of adults 85 years and over is expected to double by 2035 and nearly triple by 2060 (Vespa et al., 2020). Sarcopenia, the aging-related loss in muscle mass and function, affects up to 40% of older adults making the study of skeletal muscle health an increasingly important area of research (Mayhew et al., 2019). Innovative methodological approaches that increase the feasibility of studying skeletal muscle in older adults, particularly the very old, are critical in creating additional opportunities to address the etiology of sarcopenia and effectiveness of interventions.

Introduced in the 1960s, the Bergström biopsy (Bergstrom, 1962) is the gold standard technique for sampling skeletal muscle and has been widely used for decades in biochemical and histological studies in young and older adults. The Bergström biopsy relies on a 3–5 mm hollow needle that retrieves 25–125 mg of muscle tissue depending on whether the suction-modified approach is utilized (Bergstrom, 1975; Tarnopolsky et al., 2011). The microbiopsy (or tiny percutaneous needle biopsy) has only recently gained attention as an alternative to the Bergström for histological analysis of skeletal muscle (Pietrangelo et al., 2011; Townsend et al., 2016), despite reports of the technique going back to the late 1990s (Vescovo et al., 1996). The microbiopsy typically involves a 13- or 14-gauge (2.3 or 2 mm) needle that retrieves skeletal muscle samples via a spring-loaded instrument without the need for the 4–5 mm incision that precedes the Bergström biopsy (Pietrangelo et al., 2011; Hughes et al., 2015; Townsend et al., 2016). The microbiopsy is an appealing alternative to the Bergström given its smaller needle diameter and decreased level of invasiveness. Indeed, subjects report less pain and a preference for the microbiopsy technique compared to the Bergström (Hayot et al., 2005; Bonafiglia et al., 2020). Despite its increasing popularity, there is a paucity of work using the microbiopsy for histological analyses, which is at least partially owed to hesitation regarding the smaller tissue sample reported to range from 14 to 22 mg (Hayot et al., 2005; Hughes et al., 2015; Townsend et al., 2016). Demonstrating the efficacy of microbiopsy sampling for examining muscle fiber type morphology and composition is critical for determining the utility of this technique in the field of skeletal muscle research. Further, age-related differences in muscle fiber properties derived from microbiopsy sampling are unclear as, to the best of our knowledge, there are no reports comparing young and older adults. Age-related comparisons will indicate whether the effectiveness of the microbiopsy technique is dependent upon the population and will offer preliminary, indirect comparisons with findings derived from the Bergström biopsy.

It is expected the microbiopsy will be effective for examining aging-related changes in single fiber properties, which is useful for understanding how these alterations affect muscle health (e.g., atrophy). However, there are other important considerations when aiming to investigate the influence of age on muscle function. Older adults demonstrate reduced specific strength (muscle strength relative to muscle size), particularly at higher contraction velocities (Frontera et al., 1991; Overend et al., 1992; Jubrias et al., 1997), indicating that physiological factors other than muscle size contribute to aging-related weakness (i.e., dynapenia). The various qualitative skeletal muscle and nervous system factors and their potential role in dynapenia have received increasingly more attention over the past decade (see review, Clark and Manini, 2010) particularly due to the divergent reductions in muscle size and strength with increasing age (Goodpaster et al., 2006; Delmonico et al., 2009). Based on rodent models, impaired action potential generation via neuromuscular junction (NMJ) degradation has been suggested to have a role in aging related weakness (Padilla et al., 2021) and is believed to precede muscle fiber atrophy (Deschenes et al., 2010). However, limited evidence exists on the NMJ in humans due to inherent methodological challenges with morphological analyses. Recently, the soluble 22 kDa C-terminal agrin fragment (CAF) has been identified as an indicator of NMJ deterioration (Bolliger et al., 2010) and shows promise as a blood biomarker for sarcopenia (Hettwer et al., 2013; Pratt et al., 2021). Despite building evidence toward the association between CAF levels and muscle mass, surprisingly little work has examined its association with muscle function. In the case that NMJ deterioration augments action potential transmission to muscle fibers, muscle function may be compromised prior to substantial muscle fiber atrophy. Examining the association between NMJ degradation (as indicated by circulating CAF levels) and specific strength is critical for determining its role, if any, in maintaining muscle quality.

The microbiopsy has the potential to increase skeletal muscle research opportunities in older or fragile populations due to its reduced degree of invasiveness. It is important to determine its efficacy for analysis of muscle fiber properties in young and, in particular, older adults. The purpose of this study was to examine muscle fiber morphology and composition in young and older males using the microbiopsy sampling technique, and a secondary aim was to determine if specific strength is associated serum CAF levels in older males. In line with previous research, we hypothesize that muscle fiber composition will be similar, but type II muscle fiber size will be smaller in older males. In addition, we believe CAF levels will not be associated with specific strength in older males based on previous work indicating predominant sensitivity of CAF levels to muscle atrophy (Drey et al., 2013; Pratt et al., 2021).

## Materials and Methods

### Subjects

Thirty healthy, young (YM; *n* = 15; range = 18–26 years) and older (OM; *n* = 15; range = 66–78 years) males completed this study. Characteristics for each group are displayed in Table 1. As specified below, due to limited funds, microbiopsy samples were obtained in a subset of each group. All subjects reported not having performed structured endurance or resistance training exercise in the past 5 years and were screened for the following exclusion criteria: presence of unstable cardiovascular, metabolic or renal disease, diagnosed myocardial infarction within the last 2-years, terminal illness, a history of cerebrovascular disease, any condition affecting neuromuscular function, an artificial lower-body joint, rheumatoid arthritis, a fracture within the past year, reliance upon an assistive walking device, and Mini-mental State Exam (Folstein et al., 1975) score less than 23. Subjects were recruited from senior centers and the surrounding community through word of mouth, email, and flyer advertisements. This study was approved by the University’s Institutional Review Board prior to data collection. All subjects provided oral and written consent prior to beginning the study.

**TABLE 1:** Characteristics and CAF levels for young and older males.

Variable	Young	Older	*d*
Age (years)	20.73 ± 2.22	71.60 ± 3.94	–
Height (cm)	175.50 ± 8.55	171.08 ± 9.91	0.47
Body mass (kg)	71.03 ± 8.98	86.65 ± 16.27*	1.18
BMI (kg⋅m^–2^)	23.05 ± 2.43	29.56 ± 4.99*	1.66
Body fat %	24.65 ± 6.24	33.83 ± 6.89*	1.39
ALM (kg/BMI)	1.06 ± 0.15	0.83 ± 0.15*	1.48
Whole-muscle CSA (cm^2^)	36.21 ± 5.78	29.07 ± 4.79*	1.34
CAF (pg/ml)	369.72 ± 86.25	448.83 ± 186.61	0.53
HG strength (kg)	47.23 ± 11.56	42.45 ± 10.85	0.02

*BMI, body mass index; ALM, appendicular lean mass; CSA, cross-sectional area; CAF, C-terminal agrin fragment; HG, handgrip.*

*Indicates significant (p < 0.05) difference between groups.

### Experimental Design

Subjects visited the laboratory on two occasions separated by at least 3 days but not more than 7. Body composition assessment and familiarization with knee extensor (KE) testing was completed during the first visit. The second visit began with ultrasonography followed by KE testing and, after ∼30 min of rest (lying supine), a skeletal muscle microbiopsy. Leg dominance was determined via inquiry of preferred kicking leg (van Melick et al., 2017) and all testing was completed on the dominant leg. Subjects were instructed to avoid alcohol and vigorous physical activity for 24 and 48 h, respectively, before each visit.

Torque and velocity were recorded for the KEs using a calibrated Biodex 4 isokinetic dynamometer (Biodex Medical Systems, Inc. Shirley, NY, United States). Electromyography (EMG) of the vastus lateralis (VL) and rectus femoris (RF) was recorded using a parallel bar, bipolar surface electrode (Delsys Trigno, Delsys, Inc., Natick, MA, United States). The skin over the muscle was shaved, abraded, and cleaned with alcohol, and subsequently the electrode was placed over the muscle belly in accordance with the recommendations of the SENIAM project (Hermens et al., 1999). All signals were sampled at 2 kHz using EMGworks software (Delsys, Inc., Natick, MA, United States). Subjects were seated with hands across their chest, restraining straps over their trunk, pelvis, and thigh, and the input axis of the dynamometer aligned with the axis of rotation of the knee. Knee joint position was set at 110° (180° = full extension) for maximal voluntary isometric contractions (MVICs), while isokinetic testing involved an 80° range of motion (90°–170°).

### Blood Analysis

Venous blood was collected via venipuncture of an antecubital vein during the morning of the first visit after a 12 h fast. Serum concentrations of CAF were determined according to manufacturer guidelines in duplicate via enzyme-linked immunosorbent assay (Glory Science Co., Ltd., Shanghai, China) and microplate reader (FilterMax F5 Multi-Mode Microplate Reader, LLC, Molecular Devices, San Jose, CA, United States) at a wavelength of 450 nm. The coefficient of variation (CV%) was 5.5%. Due to the CAF value being out of the kit range for one subject, a sample size for the YM group of 14 was used for analysis.

### Body Composition

Height (cm) and weight (kg) were measured using an electronic scale (Tanita WB 3000, Arlington Heights, IL, United States). Body fat % was obtained via dual-energy x-ray absorptiometry in total body mode using a GE Lunar iDXA (GE Healthcare, Madison, WI, United States). Subsequently, appendicular lean mass (ALM) relative to body mass index was calculated using the region of interest function as suggested by Heymsfield et al. (1990). We have previously shown excellent reliability for the calculation of ALM (ICC_2,1_, SEM and CV% were 0.999, 0.042 kg⋅m^2^, and 1.2%, respectively) (Olmos et al., 2019).

Panoramic images of the VL and RF were obtained using a B-mode ultrasound (LOGIQ S7, General Electric Company, Milwaukee, WI, United States). Subjects rested in the supine position for 10 min prior to the images being captured. Images were acquired with a multifrequency linear-array probe (ML6-15 L; 5–13 MHz; 50mm field of view; General Electric Company, Milwaukee, WI, United States) using the LogiqVIEW function and following settings to ensure optimal image quality: gain = 50 dB, depth = 5.0 cm, and frequency = 12 MHz (Stratton et al., 2020). Three images of the VL and RF were captured at 50% the distance from the greater trochanter to lateral femoral epicondyle and 50% the distance from the anterior superior iliac spine to the superior border of the patella, respectively. The same investigator obtained all images while subjects were in a supine position with the knee fully extended. The investigator slowly moved the probe in the transverse plane while applying minimal and consistent pressure. Thick, double-sided tape was placed over each muscle in the transverse plane to ensure the probe was moved perpendicular to the skin. Images were scaled from pixels to centimeters prior to analysis. Cross-sectional area (CSA) of each muscle was determined using the polygon function in ImageJ software^1^ (version 1.46r, National Institutes of Health, Bethesda, MD, United States) to select as much of the muscle as possible without including the surrounding fascia. For statistical analysis, whole-muscle CSA was calculated as the sum of the VL and RF. A subset of young subjects (*n* = 11) returned for a third visit to assess test-retest reliability. Excellent reliability was shown for whole-muscle CSA (ICC_2,1_ = 0.957, SEM = 1.35 cm^2^, CV = 3.7%).

### Maximal Isometric and Isokinetic Testing

Prior to KE testing, for the purpose of characterizing sarcopenia status, handgrip strength was obtained using a dynamometer (Jamar Plus Hand Dynamometer, Patterson Medical, Cedarburg, WI, United States) while subjects were seated with the elbow joint at ∼90° and wrist in a neutral position. Subjects completed 3 trials with each hand and the highest value was used for subsequent analysis. Subjects began KE testing by performing 2 submaximal isometric contractions at 50 and 75% of perceived maximal effort. Subjects then performed 3-4 s MVICs separated by 2 min of rest. Subsequently, maximal isokinetic testing at 60°⋅s^–1^ and 180°⋅s^–1^, separated by 1–2 min of rest, was performed in a randomized order. Subjects were instructed to “kick out as *hard and fast* as possible” prior to each trial. Subjects were instructed to avoid pretension or a countermovement prior to each trial. Further, the baseline signals were visually examined after each trial to ensure adherence to instructions, and additional trials were performed as needed. Strong verbal encouragement and visual biofeedback was provided during testing.

The scaled, gravity corrected torque and velocity signals were digitally filtered with a zero lag, low-pass (40 Hz) Butterworth filter using custom written software (LabVIEW, National Instruments, Austin, TX, United States). EMG signals were processed using a 4th order Butterworth filter with a low- and high-frequency cutoff of 10 and 500 Hz, respectively, which was applied to the scaled zero means EMG signal. Peak torque (PT) was considered the highest 500 ms during MVICs, whereas it was defined as the highest 25 ms during isokinetic contractions. Specific PT was calculated via dividing the MVIC, 60°⋅s^–1^, and 180°⋅s^–1^ PT by whole-muscle CSA. EMG amplitude (RMS) for the VL and RF was obtained during the corresponding 500 ms epoch as isometric peak torque during MVICs, and throughout the entire load range for isokinetic trials at 60°⋅s^–1^ and 180°⋅s^–1^. RMS from isokinetic trials was normalized to EMG amplitude from the MVIC to serve as an indicator of muscle activation (Jenkins et al., 2015). The trial with the highest PT for all contraction types was used for subsequent analysis.

### Skeletal Muscle Microbiopsy

Microbiopsy samples were obtained from 13 YM and 11 OM. No systematic process was used for choosing the subsets that underwent a microbiopsy. Skeletal muscle samples were obtained from the lateral aspect of the VL at 50% the distance from the greater trochanter to the lateral femoral epicondyle (Hughes et al., 2015; Townsend et al., 2016). The area over the predetermined site was cleaned and disinfected with a topical antiseptic (Betadine, Stamford, CT, United States). Approximately, 2–3 ml of lidocaine (2%, without epinephrine) was injected around the area. After the area was numb, an initial incision was made to a depth of ∼2 cm via a 14-gauge pilot needle and subsequently a muscle sample was collected at a depth of ∼3 cm with a 14-gauge biopsy needle (Argon Medical, Athens, TX, United States) using an automatic biopsy instrument (Pro-Mag™ Ultra, Argon Medical, Athens, TX, United States). The biopsy needle was inserted at a 90° angle (perpendicular to the longitudinal axis of the VL) and length markers present on the needle were used to improve consistency of sampling depth. Muscle samples were obtained by the activation of a trigger button on the biopsy instrument, which unloaded the spring and engaged the needle to collect a tissue sample. Up to 4 samples (punches) were collected from the same location and stored on ice for ∼30 min. After weighing, the samples were rinsed with phosphate buffer saline (PBS) and cleaned of skin and fat tissue under a dissecting microscope, embedded in Tissue-Tek OCT compound (Sakura Finetek), flash-frozen in liquid nitrogen, and stored at –80°C. There were no complications associated with the microbiopsy for YM or OM.

### Immunofluorescence Staining

Serial 10-um transverse sections were produced with a cryostat (CryoStar NX50, Thermo Fisher Scientific) and then attached to a microscope slide (SuperFrost™ Plus, Fisher). For some subjects, 2–3 punches (separate clumps of muscle tissue) needed to be sectioned and stained to optimize fiber yield. Sections were air-dried, fixed with 3.7% formaldehyde for 10 min at room temperature (RT), permeabilized for 30 min in phosphate-buffered saline (PBS) containing 1% Triton X-100, blocked with 1% bovine serum albumin (BSA), and stained according to the multiplex staining scheme in Table 2. We used a mixture of two antibodies to invariably detect all subtypes of type II fibers, including hybrids. Fab immunoglobulin fragments were used as secondary antibodies to eliminate any possibility of non-specific cross-reactivity. Separate staining trials with each primary antibody alone were performed to confirm the specificity of the multiplex staining. All incubations were done at RT in a humidified chamber with a coverslip placed on top of staining solutions; a brief rinse in PBTx (PBS, 0.1% Triton X-100) was used between incubations. Stained slides were permanently mounted with Mowiol medium, according to previously published procedures (i.e., Rodig, 2019) and stored at 4^°^C.

**TABLE 2:** Immunofluorescence staining protocol.

Staining stages (incubation parameters)	Staining mix, (source), [titer]
Step 1 (overnight at RT)	Mouse anti-slow myosin type I IgG [clone BA-F8, Developmental Studies Hybridoma Bank (DSHB), University of Iowa, IA 52242], [1:100]
Step 2 (1 h at RT)	Goat anti-mouse IgG, Fab fragments, Alexa 488-labeled (115-547-003, Jackson ImmunoResearch), [1:400]
Step 3 (overnight at RT)	Mouse anti-fast myosin type IIA IgG (clone SC-71, DSHB) [1:100] Rabbit anti-fast myosin type II (ab91506, Abcam), [1:200]
Step 4 (1 h at RT)	Goat anti-Mouse, Cy3-labeled (115-167-003, Jackson ImmunoResearch) [1:400] Goat anti-Rabbit, Fab fragments, Alexa 647-labeled (111-607-003, Jackson ImmunoResearch) [1:400] DAPI, (D9542, Sigma) [1 ug/ml]

*RT, room temperature.*

### Laser Confocal Microscopy

Stained tissue sections were imaged using Zeiss LSM 900 system (Carl Zeiss Microscopy, LLC, White Plains, NY, United States), equipped with a 10X Plan-Neofluar objective and Zen Black software. Tiling with digital stitching was enabled to seamlessly image the entire area of each section. Stacks of optical sections spanning the entire depth of the section were flattened with the maximum intensity projections algorithm to produce the final image.

### Fiber Composition and Morphology Analysis

Boundaries of individual fibers from confocal images were manually traced using Zen software using the spline contour tool. Further analysis was conducted using ImageJ software^1^. Data for each muscle fiber was catalogued in a spreadsheet file with identification number, CSA, and the assigned fiber type. Fiber typing was conducted by a team of 3–5 operators not aware of the subject’s age and only considering the intensity of each fiber for fiber-specific myosin. Fiber type composition was calculated as the ratio of counts obtained for each fiber type to the total number of analyzed fibers from each subject. Mean CSA and CSA heterogeneity (coefficient of variation, CV) was calculated for each fiber type. Finally, as an indicator of aging-related muscle fiber remodeling (Sonjak et al., 2019), the ratio between type II and type I muscle fiber CSA for each subject was determined.

### Statistical Analyses

Normality of data was assessed via the Kolmogorov-Smirnov test. Independent samples *t*-tests were used for age-related comparisons of normally distributed variables, whereas Mann-Whitney tests were conducted for non-normally distributed variables. Levene’s test was used to test equality of variance. Associations between CAF levels, specific strength, whole-muscle CSA, and ALM were examined using Pearson product-moment correlation coefficients. Associations were only assessed in the OM group since CAF is suggested to be a biomarker of muscle health in this population. Relationships were categorized as weak, moderate, or strong relationships for correlation coefficient values of 0.35 or less, 0.36 to 0.67, and 0.68 or more (Taylor, 1990). Statistical analyses were performed using PASW software version 27.0 (SPSS Inc, Chicago, IL, United States) and an alpha level of *p* ≤ 0.05 was used to determine statistical significance. Effect size was reported using Cohen’s *d* and 0.30, 0.50, and 0.80 were used to indicate small, medium, and large effects (Cohen, 1988). All data in text and table are reported as mean ± SD, and as described below for box plots. Box plots were generated with an online resource at http://shiny.chemgrid.org/boxplotr (Spitzer et al., 2014).

## Results

### Group Characteristics and C-Terminal Agrin Fragment Levels

Characteristics and CAF levels for both groups are displayed in Table 1. OM were heavier (*p* = 0.004) and had a higher body mass index (BMI) (p < 0.001) as well as body fat % (*p* = 0.001). ALM (p < 0.001) and whole-muscle CSA (*p* = 0.001) were lower in OM, whereas CAF levels (*p* = 0.159) and handgrip strength (*p* = 0.253) were similar between groups. When VL and RF CSA were analyzed separately, VL (YM: 24.09 ± 3.52 cm^2^, OM: 18.40 ± 3.68 cm^2^; *p* < 0.001; *d* = 1.57) but not RF (YM: 12.11 ± 2.68 cm^2^, OM: 10.67 ± 2.23 cm^2^; *p* = 0.121; *d* = 0.58) CSA was lower in OM.

### Absolute and Specific Peak Torque

**FIGURE 1:**
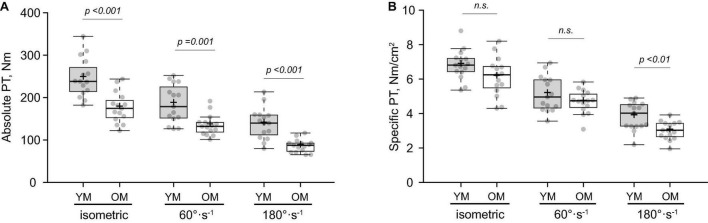
Isometric and isokinetic muscle function of the knee extensors. Absolute **(A)** and specific **(B)** peak torque (PT) in young (YM) and older (OM) males. Means and medians are represented by “+” and midlines in boxes, respectively; whiskers calculated by Tukey test. n.s., non-significant.

Absolute and specific PT data is displayed in Figure 1. Absolute PT (Figure 1A) was lower for 0°⋅s^–1^ (isometric) (p < 0.001; *d* = 1.68), 60°⋅s^–1^ (*p* = 0.001; *d* = 1.39) and 180°⋅s^–1^ (*p* < 0.001; *d* = 1.86) in OM, whereas specific PT (Figure 1B) was lower at 180°⋅s^–1^ (*p* = 0.003; *d* = 1.23) but not 0°⋅s^–1^ (*p* = 0.066; *d* = 0.69) or 60°⋅s^–1^ (*p* = 0.170; *d* = 0.51). Findings were similar when specific PT was calculated using only VL CSA. VL activation was similar between groups at 60°⋅s^–1^ (YM: 0.82 ± 0.20 % MVIC, OM: 0.95 ± 0.10 % MVIC; *p* = 0.060; *d* = 0.79) and 180°⋅s^–1^ (YM: 0.91 ± 0.23 % MVIC, OM: 0.99 ± 0.26 % MVIC; *p* = 0.400; *d* = 0.32) and the same was found for RF activation at 60°⋅s^–1^ (YM: 0.98 ± 0.16 % MVIC, OM: 0.90 ± 0.11 % MVIC; *p* = 0.130; *d* = 0.59) and 180°⋅s^–1^ (YM: 0.93 ± 0.15 % MVIC, OM: 0.90 ± 0.22 % MVIC; *p* = 0.670; *d* = 0.16).

### Composition and Morphology of Muscle Fibers

**FIGURE 2:**
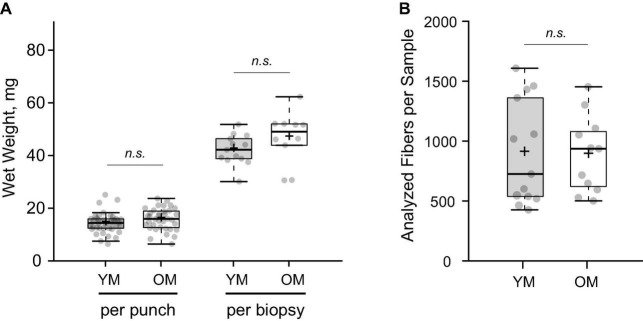
Gross parameters of muscle tissue obtained with the microbiopsy technique. **(A)** Total wet weight of tissue obtained with each individual microbiopsy sample (punch) and total per biopsy protocol (sum of punches). **(B)** Numbers of accessible muscle fibers analyzed per subject on cross sections. YM, young males; OM, older males; Means and medians are represented by “+” and midlines in boxes, respectively; whiskers calculated by Tukey test. n.s., non-significant.

**FIGURE 3:**
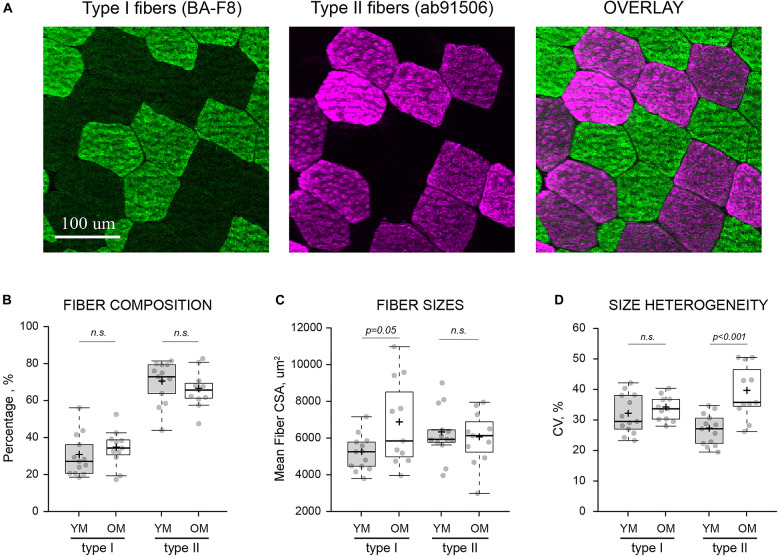
Fiber type-specific composition and morphology. **(A)** Visualization of type I and type II muscle fibers from the same area of a transversely sectioned biopsied material via multi-colored immunofluorescence and type-specific antibodies (indicated in parentheses). **(B)** Fiber type composition, expressed as the percentage of each fiber type relative to the total number of analyzed fibers. **(C)** Average cross-sectional area (CSA) of individual muscle fibers in each sample. **(D)** Heterogeneity of fiber sizes expressed as coefficient of variance (CV) for all fibers of the same type measured within each sample. YM, young males; OM, old males; Means and medians are represented by “+” and midlines in boxes, respectively; whiskers calculated by Tukey test. n.s., non-significant.

Average wet weight per microbiopsy punch was 14.39 ± 3.84 mg and 15.84 ± 4.12 mg for YM and OM (*p* > 0.05), respectively (Figure 2A). The number of fibers per cross-section was similar between YM (587.75 ± 216.58 fibers) and OM (543.27 ± 273.47 fibers) and overall, utilizing a quarter to half of the biopsied tissue, we analyzed 904.23 ± 436.31 fibers per sample and 889.15 ± 319.91 fibers per sample for YM and OM, respectively (Figure 2B). More detailed analysis of fiber type composition was performed via immunofluorescence (Figure 3A). Composition was similar for type I (*p* = 0.331; *d* = 0.39) and type II (*p* = 0.332; *d* = 0.39) fibers between YM and OM (Figure 3B). Hybrids between type I and type II fibers were extremely rare and could not be analyzed statistically. CSA was similar for type II fibers (*p* = 0.467; *d* = 0.18) between groups, however, type I fibers were larger in OM (*p* = 0.05; *d* = 0.094) (Figure 3C). OM demonstrated a smaller type II to I fiber CSA ratio (YM: 1.22 ± 0.21, OM: 0.97 ± 0.31; *p* = 0.015; *d* = 0.95). Finally, CSA was more heterogenous in OM for type II fibers (p < 0.001; *d* = 1.84) fibers, but not type I fibers (*p* = 0.343; *d* = 0.38) (Figure 3D).

### Associations

**FIGURE 4:**
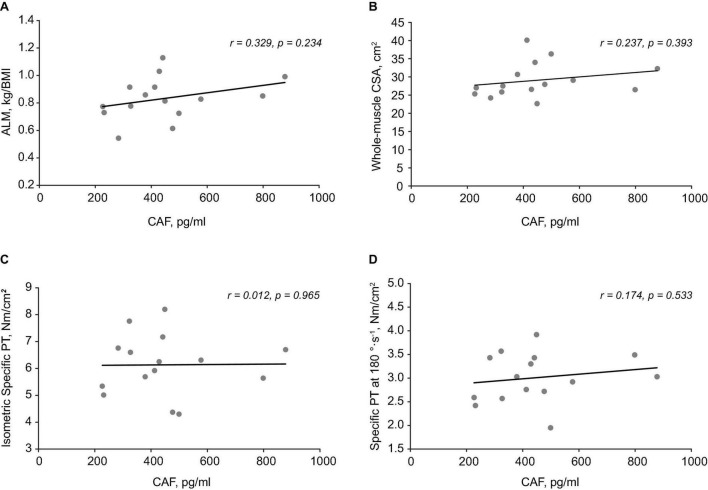
Associations between C-terminal agrin fragment (CAF) levels, body composition, and muscle function. Scatterplots demonstrating no relationship between CAF levels and appendicular lean mass (ALM) **(A)**, whole-muscle cross-sectional area (CSA) **(B)**, isometric specific peak torque (PT) **(C)**, and Specific PT at 180°⋅s^– 1^ **(D).**

C-terminal agrin fragment levels were not correlated with ALM (Figure 4A), whole-muscle CSA (Figure 4B), isometric specific PT (Figure 4C), specific PT at 180°⋅s^–1^ (Figure 4D), or specific PT at 60°⋅s^–1^ in OM. These findings remained the same when specific PT was calculated using only VL CSA. Similarly, CAF levels were not associated with absolute PT at any testing velocity (*r* = 0.213–0.379; *p* = 0.163–0.446), nor any single fiber variables (*p* > 0.05).

## Discussion

The purpose of this study was to examine muscle fiber morphology and composition in young and older males using the microbiopsy sampling technique, and a secondary aim was to determine if specific strength is associated with serum CAF levels in older males. To the best of our knowledge, this is the first study to (1) investigate the effect of age on muscle fiber properties using the microbiopsy technique and (2) assess associations between muscle quality (i.e., specific strength) and CAF levels. We demonstrated the ability to analyze hundreds of muscle fibers per subject, on par with the gold standard Bergström technique, with a relatively high proportion of type II fibers compared to previous reports using the Bergström. Our findings indicated whole-muscle atrophy in older males, but no decrements at the single fiber level. Rather, evidence of age-related remodeling was found via enlarged type I fibers and a decreased type II to I muscle fiber CSA ratio in older males. Finally, correlational evidence indicated no association between NMJ deterioration and specific strength, which was at least partially due to there being no age-related difference in CAF levels suggesting maintenance of neuromuscular integrity in our older cohort.

The capability of the microbiopsy to yield a high number of fibers, comparable to the Bergström biopsy, for histological analyses is significant. The average number of fibers analyzed across studies varies widely from 10 to 20 (Tesch et al., 1984) to a thousand (Roberts et al., 2018) per sample using the Bergström biopsy. It has been suggested that as few as 50 fibers (McCall et al., 1998) is necessary to accurately characterize the sample collected, but depending on the specific fiber type, the need to harvest up to 200 fibers has been reported (McGuigan et al., 2002). Townsend et al. (2016) analyzed ∼150 fibers on average in young males using the microbiopsy in a pilot study, but fiber yield is unclear for other studies using this technique (Hughes et al., 2015; Bonafiglia et al., 2020). We were able to analyze a few hundred fibers (>500) per microbiopsy sample in a young and older cohort, and a yield of several hundred fibers was possible by combining data from multiple cross-sections of tissue. Though combining multiple-cross sections of tissue was useful for optimizing the number of analyzed fibers, it is worth noting that a single cross-section afforded a high number of fibers (range: 188–1,059 fibers). This was despite the relatively small tissue yield compared to the Bergström, which has been the primary reason for hesitation toward microbiopsy technique. The finding that a sufficient number of fibers can be harvested for myosin heavy chain composition and histological analysis via microbiopsy presents a unique opportunity for skeletal muscle researchers. The microbiopsy has the potential to advance the study of skeletal muscle, particularly in older or fragile (e.g., sarcopenic and diseased) populations in which the increased discomfort and tissue sample size (Hughes et al., 2015; Bonafiglia et al., 2020) associated with the Bergström biopsy are a limiting factor. Future work that examines the validity of the microbiopsy technique in these population is a worthwhile endeavor. Tissue yield may even be sufficient via microbiopsy for researchers conducting multiple cellular and molecular analyses, but this is dependent on the analyses of interest and microbiopsy procedures. For example, we extracted ∼45 mg of tissue from three microbiopsy punches, whereas Hughes et al. (2015) retrieved ∼80 mg (and up to 120 mg) after 5 punches. Further, due to the well documented heterogeneity of muscle fiber types (Lexell and Taylor, 1989a,b; Horwath et al., 2021), it is recommended that more than one biopsy be conducted per visit (Lexell and Taylor, 1989b; Horwath et al., 2021) which is more feasible with the microbiopsy given it is minimally invasive.

Muscle fiber CSA values were similar to several Bergström studies (Verdijk et al., 2007; Nilwik et al., 2013; Horwath et al., 2021). The present study found no evidence of age-related muscle fiber atrophy, and while similar findings have been reported for type II muscle fibers in males (Trappe et al., 2003; Roberts et al., 2018), this is in disagreement with consistent reports of preferential type II muscle fiber atrophy (Lexell et al., 1988; Walker et al., 2012; Nilwik et al., 2013). This finding is at least partially due to our older sample being healthy and non-sarcopenic as indicated by ALM (kg/m^2^) and handgrip strength values (Cruz-Jentoft et al., 2019). More specifically, only two older males had an ALM less than 7.0 kg/m^2^ while none were below the threshold for handgrip strength (27 kg), thus sarcopenia was not present in our older sample. The increased type II to I muscle fiber CSA ratio was driven by enlarged type I muscle fibers, and not atrophied type II fibers, in older males. These findings likely characterize compensatory changes at the cellular level (Aniansson et al., 1992; Frontera et al., 2008) that contribute to the healthy, non-sarcopenic status of the older males. As suggested by Roberts et al. (2018), the greater heterogeneity in CSA for type II fibers may indicate both, hypertrophic and atrophic responses in individuals from the older male group. Whole-muscle atrophy, of the VL in particular, was present in older males, which given the lack of muscle fiber atrophy, suggests that the loss of muscle fibers was influential for this finding (Lexell et al., 1988; Frontera et al., 2000). Composition of muscle fiber type was similar between age groups, which is in line with previous studies (Lexell et al., 1988; Snijders et al., 2014; Sonjak et al., 2019). Generally, most studies using the Bergström biopsy report proportions of 40–50% for type I and type II fibers, but we found type II fiber composition to be 69% and 65% in young and older males, respectively. Our type II fiber composition data is similar to two other studies using the microbiopsy (Hayot et al., 2005; Hughes et al., 2015). Hughes et al. (2015) directly compared the microbiopsy and Bergström technique and found a higher proportion of type IIx fibers with the microbiopsy. The relatively large proportion of type II fibers is noteworthy and may be attributed to more superficial sampling using the microbiopsy (Lexell et al., 1983). Indeed, in consideration of the smaller tissue sample yield from the microbiopsy, the use of ultrasonography for needle guidance (Jandova et al., 2020) should be considered for future work to ensure consistent depth of muscle sampling in young and older subjects as age-related changes in subcutaneous fat are expected. Importantly, the novel data in the present work provides a descriptive age-related comparison using the microbiopsy, but more work is needed to further elucidate if age-related differences in fiber type composition and histology are dependent up sampling technique. Follow-up work directly comparing muscle fiber properties using the Bergström and microbiopsy (with special attention to sampling depth) in young and older adults is required to verify the level of agreement between the techniques.

Similar to previous research, we showed that specific strength was only decreased at a higher contraction velocity (180° s^–1^) (Frontera et al., 1991; Overend et al., 1992; Jubrias et al., 1997) in older males. This suggest that qualitative factors are influential for the age-related loss of strength, particularly during a higher contraction velocity. Atrophy or preferential loss of type II fibers were not responsible as neither were present in our older sample. Decreased tendon stiffness in older males (Quinlan et al., 2017) cannot be ruled out and could be expected to diminish force transmission at higher contraction velocities (Hauraix et al., 2013). We conducted an inquiry on the association between NMJ degradation (as indicated by CAF levels) and specific strength. CAF levels were not associated with specific strength at any velocity, nor whole-muscle CSA or ALM in older males. It is unclear what contributed to the age-related decrease in specific strength at 180° s^–1^ but changes at the molecular level such as slowed cross-bridge kinetics could be responsible (Höök et al., 2001; Miller et al., 2013). These findings are in contrast with recent studies demonstrating a negative relationship between CAF levels and lean mass (Drey et al., 2013; Pratt et al., 2021). The size and composition of our sample were likely influential for these findings as previous studies were conducted in larger, more heterogenous populations (e.g., greater age range). Indeed, given the non-sarcopenic phenotype of our older sample, the moderate effect size though non-significant result for CAF levels between age groups is not overly surprising. This is because CAF levels appear to be influenced by sarcopenia status and not aging *per se* (Hettwer et al., 2013; Olmos et al., 2019). Accordingly, the finding that CAF levels were not associated with specific strength supports the concept that this biomarker is particularly sensitive to muscle wasting (Pratt et al., 2021) and less so to other physiological factors influencing strength. Nevertheless, evidence on CAF levels as an indicator of NMJ degradation and the role this plays in distinguishing sarcopenia severity is still in its infancy, thus these findings contribute to the understanding of this biomarker as an emerging biomarker for sarcopenia.

There were a few limitations associated with the current study. Our findings only characterize males, which is an important consideration since there are sex differences associated with aging-related changes in whole-muscle and single fiber size (Reid et al., 2014; Roberts et al., 2018). Particularly for findings derived from the microbiopsy, our sample size was limited and may not have possessed the necessary statistical power for between groups analyses. Despite our muscle fiber CSA values being similar to previous data obtained from the Bergström biopsy (Verdijk et al., 2007; Nilwik et al., 2013; Horwath et al., 2021), it is possible the 30-min delay in freezing samples affected muscle fiber size. Given the post-biopsy procedures were identical for all subjects and sample wet weight was similar between young and older males, it is unlikely any influence of the 30-min delay was age dependent. While isopentane is often used as an intermediate coolant, a potential advantage of the microbiopsy method is its compatibility with a simple flash-freezing technique. Upon analyzing hundreds of cryosections, we did not detect systemic presence of freezing artifacts, which suggests that smaller-than-typical muscle tissue volume receives adequate cooling rates when plunged directly into liquid nitrogen (Lee et al., 2020). Finally, while it was not the purpose of this study to directly compare the microbiopsy and Bergström techniques, the lack of a comparison could be considered a limitation. Indirect comparisons based on previous studies using the Bergström technique were emphasized with the intention to provide a preliminary perspective and bring attention to the need for comparing the techniques for examining aging-related changes in muscle fiber properties.

The present study demonstrated, for the first time, the efficacy of the microbiopsy sampling technique to be used for analysis of muscle fiber morphology and composition in older adults. We showed that the microbiopsy can yield more than a sufficient number of fibers for histological analyses, which suggest the microbiopsy is a viable muscle sampling alternative to the Bergström for researchers wanting to minimize the challenges associated with the more invasive Bergström technique. Further, as human lifespan continues to increase and the study of skeletal muscle health in advanced age (i.e., ≥ 8th decade) becomes increasingly relevant, we posit that the microbiopsy technique will be critical in the advancement of therapies for improving quality of life. With regard to our age-related findings, no muscle fiber atrophy or changes in composition were found but enlarged type I fibers were present in older males. These findings were at least partially due to our high-functioning older sample and indicate healthy aging. No association was found between a relatively new biomarker of neuromuscular junction degradation (circulating C-terminal agrin fragment levels) and whole-muscle CSA, appendicular lean mass, or specific strength. These findings in conjunction with those from others suggest that aging-related deterioration of the neuromuscular junction may primarily contribute to muscle wasting and not necessarily muscle function.

## Data Availability Statement

The raw data supporting the conclusions of this article will be made available by the authors, without undue reservation.

## Ethics Statement

The studies involving human participants were reviewed and approved by Kennesaw State University Institutional Review Board. The patients/participants provided their written informed consent to participate in this study.

## Author Contributions

GH and ALB contributed to study conceptualization and design, supervision, data processing, statistical analysis, data interpretation, and writing of the manuscript. TV contributed to the study conceptualization and design, supervision, data processing, and editing of the manuscript. PH, AO, BD, ARB, KK, MJ, SK, and SA contributed to data collection, data processing, and editing of the manuscript. All authors contributed to the article and approved the submitted version.

## Conflict of Interest

The authors declare that the research was conducted in the absence of any commercial or financial relationships that could be construed as a potential conflict of interest.

## Publisher’s Note

All claims expressed in this article are solely those of the authors and do not necessarily represent those of their affiliated organizations, or those of the publisher, the editors and the reviewers. Any product that may be evaluated in this article, or claim that may be made by its manufacturer, is not guaranteed or endorsed by the publisher.

## Footnotes

1
https://imagej.nih.gov
